# Comparative Study of Decision-Level Fusion Strategies for Multi-Sensor CNN-Based Bearing Fault Diagnosis

**DOI:** 10.3390/e28091020

**Published:** 2026-09-12

**Authors:** Iman Makrouf, Mourad Zegrari, Khalid Dahi, Demba Diallo, Meryem Abtane, Ilias Ouachtouk

**Affiliations:** 1 Digital Engineering for Leading Technology and Automation, National Higher School of Arts and Crafts Casablanca, Hassan II University of Casablanca, Casablanca 20670, Morocco; 2 Complex Systems and Interactions, Ecole Centrale Casablanca, Bouskoura 27182, Morocco; khalid.dahi@centrale-casablanca.ma (K.D.); meryem.abtane@centrale-casablanca.ma (M.A.); 3 GeePs, CNRS, CentraleSupélec, Université Paris-Saclay, 91190 Gif-sur-Yvette, France

**Keywords:** decision-level fusion, multi-sensor fusion, convolutional neural network, Dempster–Shafer theory, noise stress

## Abstract

Bearing fault diagnosis increasingly relies on multiple sensors, since compound or multi-location faults can produce signatures that are only partially captured by a single sensor. The decision-level fusion (DLF) of independently trained models offers a practical way to combine such complementary information, yet systematic comparisons across DLF techniques remain scarce, particularly for measurements from different sensor locations. This paper benchmarks six DLF strategies, i.e, Max, Average, Majority Voting, Weighted Sum, Dempster–Shafer, and Stacking, on a dual-branch one-dimensional convolutional neural network (1D-CNN) with each branch trained end-to-end on vibration signals from a distinct bearing location. On a two-sensor test bench covering seven health conditions, all methods exceed 99.7% accuracy on clean signals, while Dempster–Shafer fusion proves markedly more robust under noise, retaining up to 84% accuracy at a 5 dB signal-to-noise ratio (SNR). A conflict-coefficient analysis further provides an interpretable account of when fusion succeeds, linking performance to the confidence complementarity between branches.

## 1. Introduction

Rolling element bearings are among the most failure-prone components in rotating machinery, and their degradation directly affects the safety, availability, and operational cost of industrial systems ranging from manufacturing lines to power generation and transportation [1,2]. An undetected bearing defect can lead to severe downtime or catastrophic failure, which makes early and reliable fault diagnosis such a critical task. Bearing fault diagnosis has evolved firstly through signal processing methods relying on handcrafted features, including the analysis of vibration, acoustic, or current signals in the time domain, frequency domain, and time–frequency domain [3,4]. Signal processing provides a good understanding and analysis of fault signatures in acquired signals; however, it still has the shortcomings of handcrafted features and expert engineering knowledge for signal analysis, which limit the automation of fault diagnosis [5]. Thus, shallow machine learning has been widely used [6], in which classifiers are trained on handcrafted features requiring expert knowledge; while effective, these approaches still limit fully automated diagnosis and lack robustness under complex fault conditions. More recently, in an end-to-end fault diagnosis framework, deep learning approaches learn discriminative representations directly from raw vibration signals. Convolutional neural networks (CNNs) [7], in particular, have become the dominant architecture for this task, owing to their ability to capture local, translation-invariant temporal patterns without manual feature engineering. However, industrial systems often exhibit multiple, compound, or coupled faults occurring simultaneously, whose signatures overlap and interact in complex ways that a single sensor cannot fully capture [8]. This limitation makes multi-sensor information fusion a crucial improvement, as combining complementary information from sensors placed at different locations enhances fault diagnosis performance and reliability.

Several recent works have adopted multi-sensor fusion to exploit the volume of data generated by modern multi-sensor industrial systems [9]. According to a recent fusion taxonomy [10], fusion can be applied at three levels: the input level, model level, and decision level. Input-level fusion, also referred to as feature-level fusion, combines multiple data modalities or features before processing; at this level, Yan et al. [11] fused multichannel time–frequency representations into a single colored image, which is then input to an attention-based residual CNN model for effective fault diagnosis, while Makrouf et al. [12] used a continuous wavelet transform (CWT)-based vibration–acoustic fusion scheme that uses a Maximum Energy-to-Shannon-Entropy-Ratio criterion to fine-tune pre-trained CNNs under variable operating speeds. Model-level fusion joins information through shared layers or cross-branch interactions during training; for instance, Li et al. [13] proposed an MSIF-Convformer that uses parallel CNN encoders with a cross-attention mechanism to fuse multi-source time–frequency representations, while Ye et al.’s [14] method integrates a global interactive perception module to fuse acoustic and vibration features directly within the network. At the highest level of fusion, decision-level fusion combines the independent outputs, probabilities, or belief masses produced by separately trained models into a single final decision: it could be an output fusion driven from different sensors or from different models at one sensor. For example, at the decision-level fusion (DLF) stage, Xu et al. [15] addressed diagnostic trustworthiness under distribution shift by proposing a multi-scale probabilistic neural network combined with three uncertainty quantification-based decision fusion strategies, i.e., hard voting, soft voting, and Bayesian causal inference; their proposed framework increases confidence for known fault types but also increases uncertainty for unknown ones. Chao et al. [16] combined the class-probability outputs of three CNN branches, each processing one vibration channel, through a learned weight matrix to improve leakage-diagnosis accuracy over single-sensor baselines for an axial piston pump. Xu et al. [17] combined the pre-diagnoses of parallel base models through a dynamically enhanced weighted voting strategy that assigns each model’s vote a weight derived from its accuracy and misclassification behavior.

Each fusion level trades information richness against deployability. Input-level fusion preserves the most raw information but demands consistent data structures across sensors and often requires architecture redesign for each new sensor combination [1,18]. Model-level fusion lowers computational cost and benefits from advances in deep architectures, but its shared training pathway lets early-stage errors propagate into the fused representation [10]. Decision-level fusion avoids this propagation, since combined outputs come from independently trained base models whose errors are largely uncorrelated; together with its low data requirements and minimal hardware overhead, this makes it the most favored for practical deployment [19]. This conclusion is also reached from a purely industrial-deployment standpoint [10], where decision-level fusion adds negligible hardware, labeling, or real-time cost precisely because fusion happens only after each branch has already produced its output. Its performance ceiling is therefore set by the diversity and independence of the fused decisions rather than by deployment cost, making it particularly well suited to small-scale, heterogeneous, and sensor configurations at different locations.

Within DLF, various techniques have been reported in the literature, including voting rules [7], ensemble-learning fusion methods such as Stacking [20] and Boosting [21], and Dempster–Shafer theory [22]. Other competitive fusion strategies have also been explored at both the feature (early) and decision (late) levels, including copula-based fusion, Behavior Knowledge Space (BKS), and α-integration. The latter provides an optimal solution under the minimum probability of error (MPE) and least mean-square error (LMSE) criteria, and it has recently been extended to incorporate graph regularization and multiclass classification [23]. However, existing works [19,22,24] do not provide a systematic comparative analysis across such techniques, even though the choice of fusion rule can significantly affect the final diagnosis outcome. This gap is particularly relevant in multi-location fault scenarios, such as axle-bogie train bearings [25], which represent a practical case of multi-location sensing that we emulate here using a two-bearing, two-sensor laboratory setup with vibration measurements acquired from sensors mounted at different bearing locations. In this setting, decision-level fusion offers particular practicality and interpretability, since location-specific decisions can be combined and examined directly. Despite its relevance to this complex and industrially important scenario, such a setting remains insufficiently investigated in the existing fusion literature. In particular, prior studies employing Dempster–Shafer (DS) theory primarily use it as the proposed fusion solution rather than systematically evaluating it against alternative decision-level fusion rules, and they do not provide an analysis of the conditions under which fusion succeeds or fails at the sample level. This paper therefore focuses on a systematic comparative benchmark of six decision-level fusion techniques, Max, Average, Majority Voting, Weighted Sum, Dempster–Shafer, and Stacking, using a dual-branch end-to-end 1D-CNN multi-sensor architecture and evaluating their performance under different noise levels and fault conditions. This paper covers both simple and compound fault scenarios occurring at different bearing locations and captured from different measurement points. To interpret the comparative results, a conflict-based analysis is further conducted using the Dempster–Shafer conflict coefficient together with branch-level confidence analysis, revealing how complementary sensor confidences can benefit fusion and identifying conditions under which high inter-branch conflict limits fusion performance.

The remainder of this paper is organized as follows: Section 2 reviews the theoretical background. Section 3 presents the proposed framework and experimental setup. Section 4 reports and discusses the results, and Section 5 outlines the conlusion.

## 2. Theoretical Background

### 2.1. One-Dimensional Convolutional Neural Network

Vibration signals are inherently sequential, and 1D convolutional neural networks are well suited to this structure where convolutional filters slide along the temporal axis and learn local, translation-invariant patterns directly from raw waveforms without any handcrafted preprocessing step [26]. Stacking several convolutional blocks lets the network build a hierarchy of representations from short transient patterns in early layers to more abstract, fault-specific signatures in deeper ones.

Formally, given an input signal $x\in R^{L}$, a convolutional layer applies a learnable kernel $w\in R^{k}$ along the temporal dimension to produce a feature map:

(1) $$h_{j}=\sigma \left(\sum_{i}w_{i}\cdot x_{j+i}+b\right)$$

where *b* is a bias term and σ(·) denotes the rectified linear unit (ReLU) nonlinearity. Max-pooling layers interleaved between convolutional blocks progressively reduce the temporal resolution while reinforcing invariance to small time shifts. After the last convolutional block, the resulting feature map is flattened and passed through fully connected layers, ending in a softmax layer that turns the network’s logits into a probability distribution over the *C* candidate fault classes:

(2) $$p\left(y=c|x\right)=\frac{\exp(z_{c})}{\sum_{c^{\prime }=1}^{C}\exp\left(z_{c^{\prime }}\right)}$$

with $z_{c}$ the logit associated with class *c*. Training proceeds by minimizing the categorical cross-entropy loss through backpropagation. In this paper, each branch of the diagnostic framework is one such 1D-CNN, which is trained independently on the vibration signal of a single sensor; the resulting per-branch class probabilities are the raw material that the decision-level fusion strategies described next operates on.

### 2.2. Decision-Level Fusion Strategies

Decision-level fusion combines the independent outputs of already trained classifiers rather than their raw signals or intermediate features. Because fusion is applied after each classifier has reached its own decision, the combination rule is independent of the classifiers themselves, it can be changed to suit the application without retraining the classifiers, and it can combine models built on different sensors or architectures without requiring them to share a common representation [27].

Formally, let $p_{m,c}$ denote the posterior probability assigned to class c∈{1,…,C} by branch m∈{1,…,M}. Decision-level fusion maps these *M* independent posterior vectors to a single fused decision $\hat{y}$. Six representative strategies are compared here, covering the main fusion families reported in the literature [24]: fixed rules (Max, Average, Majority Voting), a reliability-weighted rule (Weighted Sum), an evidence-theoretic combiner (Dempster–Shafer), and a learned combiner (Stacking), which are presented in order of increasing reliance on branch-level confidence and adaptivity.

#### 2.2.1. Max Rule

The predicted class is the one receiving the single highest confidence score across all branches:

(3) $$\hat{y}=\arg\max_{c}\;\max_{m}\;p_{m,c}$$

This rule effectively defers the decision to whichever model is most confident at a given instant, which is useful when one sensor is reliably more informative than the other but harmful if that confidence is misplaced.

#### 2.2.2. Average Rule

The Average rule computes the class-wise mean of posterior probabilities and selects the highest-scoring class:

(4) $$\hat{y}=\arg\max_{c}\frac{1}{M}\sum_{m=1}^{M}p_{m,c}$$

By averaging, individual classifier uncertainties are smoothed out, making this rule effective when classifiers are of comparable accuracy.

#### 2.2.3. Majority Voting

Each classifier independently produces a hard prediction ${\hat{y}}_{m}=\arg\max_{c}\;p_{m,c}$, and the final decision is the class receiving the most votes:

(5) $$\hat{y}=\arg\max_{c}\sum_{m=1}^{M}1\left[{\hat{y}}_{m}=c\right]$$

where 1[·] is the indicator function. While simple and robust to individual errors, this rule discards the confidence information encoded in posterior probabilities.

#### 2.2.4. Weighted Sum

The Weighted Sum rule extends the Average rule by assigning a weight $\alpha_{m}$ to each classifier proportional to its validation accuracy:

(6) $$\hat{y}=\arg\max_{c}\sum_{m=1}^{M}\alpha_{m}\cdot p_{m,c},\;\mathrm{where}\;\sum_{m=1}^{M}\alpha_{m}=1$$

(7) $$\alpha_{m}=\frac{{\mathrm{Acc}}_{m}}{\sum_{k=1}^{M}{\mathrm{Acc}}_{k}}$$

This ensures stronger classifiers contribute more to the final decision, while equal accuracies recover the Average rule as a special case.

#### 2.2.5. Dempster–Shafer Theory of Evidence

The Dempster–Shafer Theory (DST) [28] is a mathematical framework for reasoning under uncertainty that assigns belief to composite hypotheses rather than individual events, making it well suited for combining uncertain decisions from multiple independent sources.

**Frame of Discernment.** The monitored system is assumed to be in one of *C* possible states, i.e, from healthy to the different fault types, forming the frame of discernment:

(8) $$\Theta =\{c_{1},c_{2},\ldots ,c_{C}\}$$

Its power set $2^{\Theta }$ contains all possible subsets of Θ, including the empty set Ø and Θ itself. Any element $A\in 2^{\Theta }$ is referred to as a proposition.

**Basic Belief Assignment (BBA).** A mass function $m:2^{\Theta }\to \left[0,1\right]$ satisfies m(Ø)=0 and $\sum_{A\in 2^{\Theta }}m\left(A\right)=1$. In the proposed framework, the BBA of the model *i* is constructed directly from its softmax output $p_{i,c}$ over the *C* fault classes:

(9) $$m_{i}\left(c_{k}\right)=p_{i,k},\;k=1,\ldots ,C$$

where $m_{i}\left(\Theta \right)$ represents the residual ignorance that model *i* cannot commit to any specific class. Thus, it is representing the portion of belief left uncommitted to any specific class as distinct from disagreement between particular classes. This allows DST to formally separate genuine uncertainty about the true class from conflict among specific hypotheses.

In principle, $m_{i}\left(\Theta \right)$ can be set in different ways: as a fixed value common to all samples, as a quantity derived from model confidence (e.g., proportional to $1-\max_{k}p_{i,k}$, so that less confident predictions leave more mass unassigned), or set to zero when the classifier’s output is treated as already exhausting the available belief. In this paper, we adopt the latter setting, $m_{i}\left(\Theta \right)=0$, since the softmax output already satisfies $\sum_{k=1}^{C}p_{i,k}=1$, leaving no mass for Θ and populating no composite hypothesis.

**Dempster’s Rule of Combination.** For *M* independent BBAs, the fused mass is obtained by successive application of the orthogonal sum ⊕:

(10) $$m_{\mathrm{fused}}=m_{1}⊕m_{2}⊕\cdots ⊕m_{M}$$

where for any two BBAs $m_{i_{1}}$ and $m_{i_{2}}$:

(11) $$m\left(A\right)=\left(m_{i_{1}}⊕m_{i_{2}}\right)\left(A\right)=\left\{\begin{array}{cc} \frac{m_{12}\left(A\right)}{1-K}, & A\neq Ø\\ 0, & A=Ø \end{array}\right.$$

with the conjunctive consensus and conflict factor defined as

(12) $$\begin{array}{cc} m_{12}\left(A\right) & =\sum_{\begin{array}{c} B_{1},B_{2}\in 2^{\Theta }\\ B_{1}\cap B_{2}=A \end{array}}m_{i_{1}}\left(B_{1}\right)\;m_{i_{2}}\left(B_{2}\right)\\ K & =\sum_{\begin{array}{c} B_{1},B_{2}\in 2^{\Theta }\\ B_{1}\cap B_{2}=Ø \end{array}}m_{i_{1}}\left(B_{1}\right)\;m_{i_{2}}\left(B_{2}\right) \end{array}$$

where *K* quantifies inter-source conflict; the rule requires K<1 to avoid counterintuitive results [28].

**Final Decision.** The diagnostic output is the class with the highest fused mass:

(13) $$\hat{y}=\arg\max_{c_{k}\in \Theta }\;m_{\mathrm{fused}}\left(c_{k}\right),\;k=1,\ldots ,C$$

#### 2.2.6. Stacking

Stacking treats fusion itself as a learning problem: a meta-learner is trained to map the concatenated classifiers outputs to the true class label. The meta-learner’s input is the stacked probability vector

(14) $$z=\left[p_{1,1},\ldots ,p_{1,C},p_{2,1},\ldots ,p_{M,C}\right]\in R^{M\times C}$$

fitted on a held-out validation set, using a logistic regression, a support vector machine (SVM), or a shallow neural network as the combining function [29]. Unlike the fixed rules above, stacking can capture nonlinear interactions between branches and adapt to different classifier performance.

## 3. Proposed Diagnostic Framework

**Figure 1:**
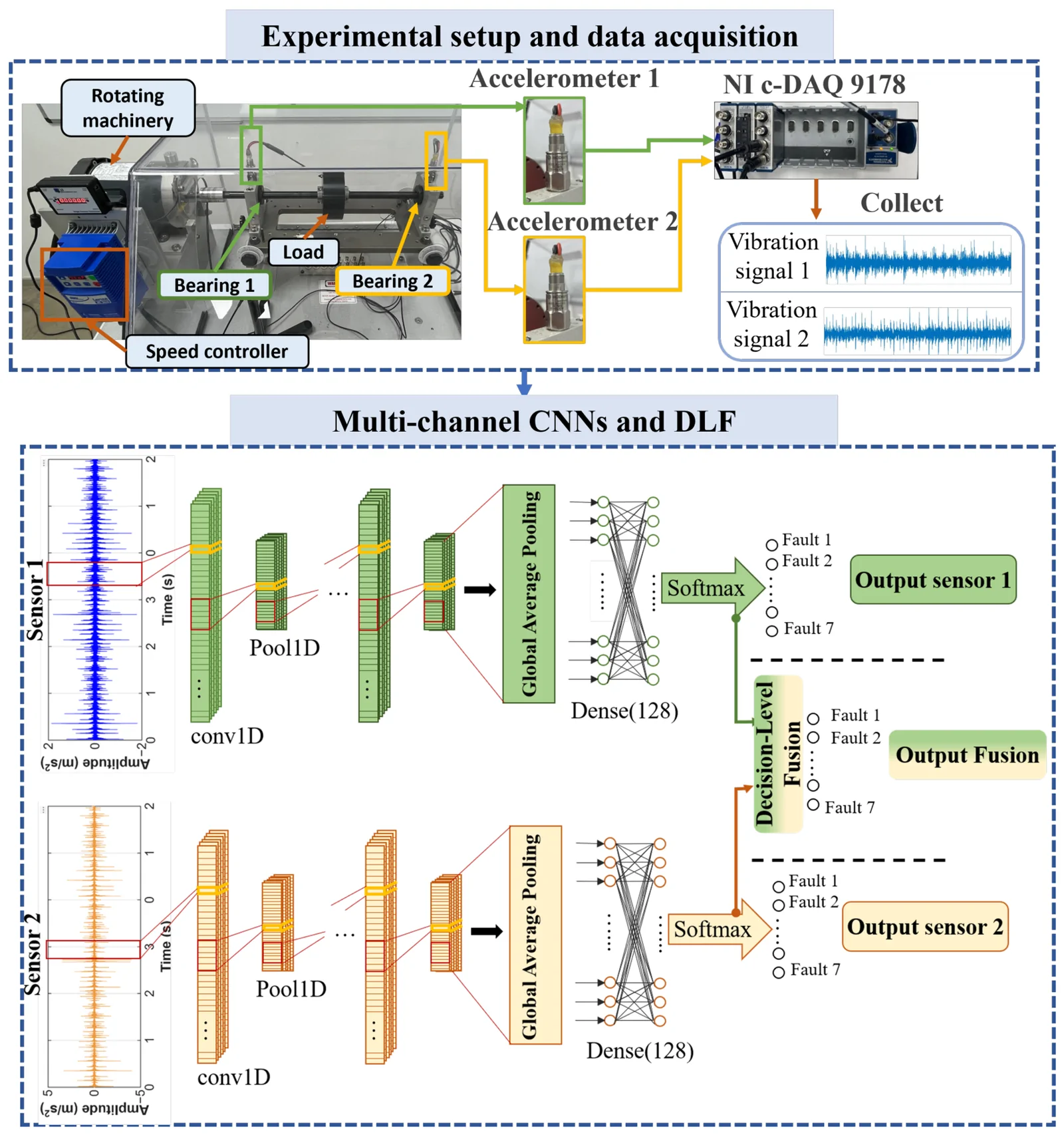
Proposed diagnostic framework: multi-channel CNNs with decision-level fusion.

The proposed framework uses the decision-level fusion described in Section 2.2 around two 1D-CNN models in a dual-branch architecture, which are each dedicated to a single sensor location. CNN Branch 1 and Branch 2 are trained separately on the vibration signals of their respective bearing position; each branch produces its own class probability vector through a softmax output layer. The probability vectors obtained from each CNN model are combined together through one of the six fusion strategies presented above to produce the final decision $\hat{y}$. The overall architecture, depicted in Figure 1, is designed to provide per-branch modular learning, allowing each CNN branch to be trained, validated, and upgraded independently, with fusion applied at inference time.

### 3.1. Experimental Setup and Data Acquisition

Data were collected on a SpectraQuest Machinery Fault Simulator (MFS) hosted at the Complex Systems and Interactions (CSI) Laboratory, Ecole Centrale Casablanca, which is a test bench built to reproduce realistic rotating-machinery faults under controlled operating conditions. The bench consists of a variable-speed motor driving a shaft supported by two rolling-element bearings, denoted Bearing 1 and Bearing 2, each instrumented with a PCB 352A21 piezoelectric accelerometer (10 mV/g sensitivity) mounted on the bearing housing in the axial direction, as shown in Figure 1. Throughout this paper, Sensor 1 refers to the accelerometer mounted on Bearing 1, and Sensor 2 refers to the accelerometer mounted on Bearing 2, with each sensor feeding the corresponding CNN branch (CNN-1 and CNN-2, respectively). Both accelerometers were sampled synchronously at 25,600 Hz through a National Instruments cDAQ-9178 chassis interfaced with LabVIEW, ensuring that the two sensor streams remain time-aligned for every acquisition.

Seven health conditions were investigated, as summarized in Table 1. Three single-location bearing faults were induced on Bearing 1 with a ball fault (BA), an outer race fault (OR), and an inner race fault (IR), leaving Bearing 2 free of any localized defect. A combined defect (CBD) reproduces all three fault types simultaneously on Bearing 1. In every case, vibration energy propagates mechanically through the shaft and housing structure, so both sensors register some signature of the fault regardless of its physical origin. A dedicated multi-location condition, denoted IROR, combines an outer race fault on Bearing 1 with an inner race fault on Bearing 2. Shaft unbalance (UNB) is induced through an eccentric disc, while the healthy condition (NO) corresponds to normal operation with no induced defect. Experiments were repeated at three rotational frequencies (10, 15, and 20 Hz) under two load conditions (no load and 5 kg), yielding 540 signals of 76,800 samples per condition and per sensor, for a total of 3780 signals per branch. The dataset was split at the signal level into training, validation, and test subsets following a 60:20:20 ratio. This split was applied to the 540 raw signals per condition prior to the sliding-window segmentation described in Section 3.2: all N=149 overlapping segments generated from a given raw signal are therefore assigned entirely to a single subset (train, validation, or test), preventing any segment-level leakage between subsets despite the 50% window overlap.

**Figure 2:**
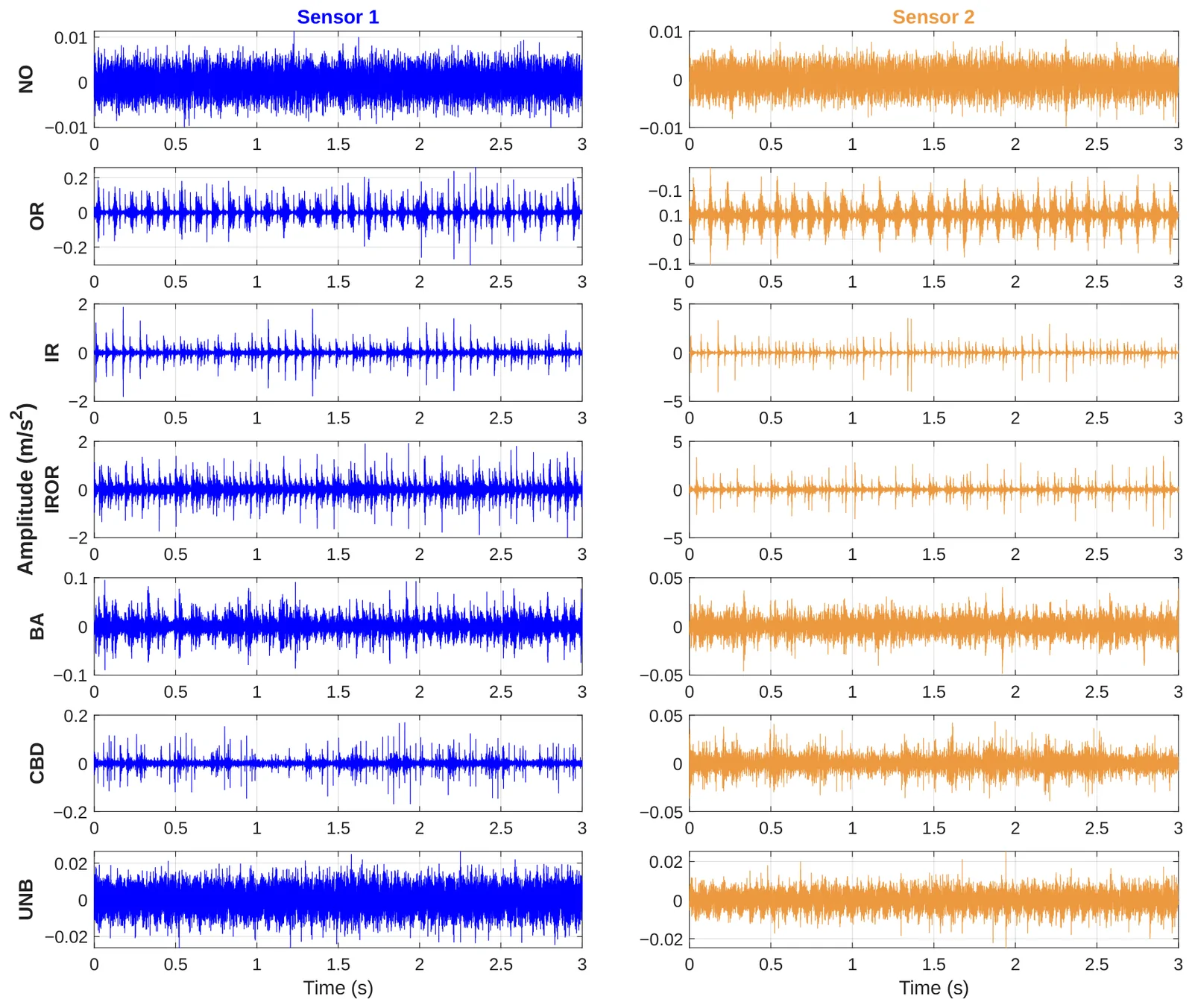
Time-domain vibration signals acquired from Sensor 1 and Sensor 2 for each of the seven health conditions at a rotational speed of 10 Hz. NO: healthy (no induced defect); BA: ball fault; OR: outer race fault; IR: inner race fault; CBD: combined bearing defect (BA + OR + IR on Bearing 1); IROR: combined inner and outer race fault at two distinct locations (outer race fault on Bearing 1, inner race fault on Bearing 2); UNB: shaft unbalance.

Representative time-domain signals acquired from both sensors for each of the seven health conditions are shown in Figure 2. Localized defects (BA, OR, IR, CBD, IROR) produce clearly visible periodic impulsive transients superimposed on the background vibration, with amplitude and repetition pattern varying by fault type and location, whereas the healthy (NO) and unbalance (UNB) signals remain comparatively smooth and dominated by broadband stochastic content.

### 3.2. Signal Segmentation and 1D-CNN Architecture

Each raw vibration signal contains 76,800 data points, which is too long to be used directly as a single network input and yields only one training example per acquisition. To address this, every signal is partitioned into fixed-length segments using a sliding window both to produce inputs of a size compatible with the CNN and to substantially increase the number of training instances available from a limited number of acquisitions. This procedure yields N=149 fixed-length, partially overlapping segments per signal, each fed directly to the 1D-CNN without any handcrafted feature extraction, allowing the network to learn discriminative temporal patterns end to end from the raw waveform. The 50% overlap ensures that fault-related transients occurring near a window boundary are still fully captured within at least one adjacent segment while also acting as a form of data augmentation. As noted in Section 3.1, segmentation is applied *after* the signal-level train/validation/test split, so overlapping segments from a given signal never cross subset boundaries.

Each segment $x^{\left(k\right)}\in R^{1024}$ is then processed by the 1D-CNN summarized in Table 2. The network consists of four successive convolutional blocks, which are each composed of a Conv1D layer, batch normalization (BN), a ReLU activation, and a max-pooling operation, which together progressively extract and downsample local temporal features while increasing the number of feature channels (32→64→128→256). The resulting feature maps are reduced by a global average pooling layer into a compact 256-dimensional representation, which is passed through a fully connected layer of 128 units with dropout regularization before the final softmax layer produces a probability distribution over the seven fault classes. The complete architecture totals 169,063 trainable parameters.

## 4. Results and Discussion

All experiments were conducted on the Kaggle cloud platform using an NVIDIA Tesla P100 graphics processing unit (GPU) (16 GB HBM2, 3584 CUDA cores, 9.3 TFLOPS). Both CNN models were trained independently using the Adam optimizer with a learning rate of 0.001, a batch size of 256, and a maximum of 50 epochs with an early stopping criterion applied upon no improvement in validation loss for five consecutive epochs. For the Stacking fusion strategy, the meta-learner classifier is implemented as a support vector machine (SVM) with a radial basis function (RBF) kernel (C=10, gamma=scale), which was trained via scikit-learn. Its input is the 14-dimensional vector obtained by concatenating the seven-class posterior probabilities produced by CNN-1 and CNN-2 on the validation set with the corresponding ground-truth label as the target. The meta-learner is trained once, on clean (noise-free) validation-set predictions, and applied unchanged to the test set across all SNR levels.

### 4.1. Individual CNN Performance

**Figure 3:**
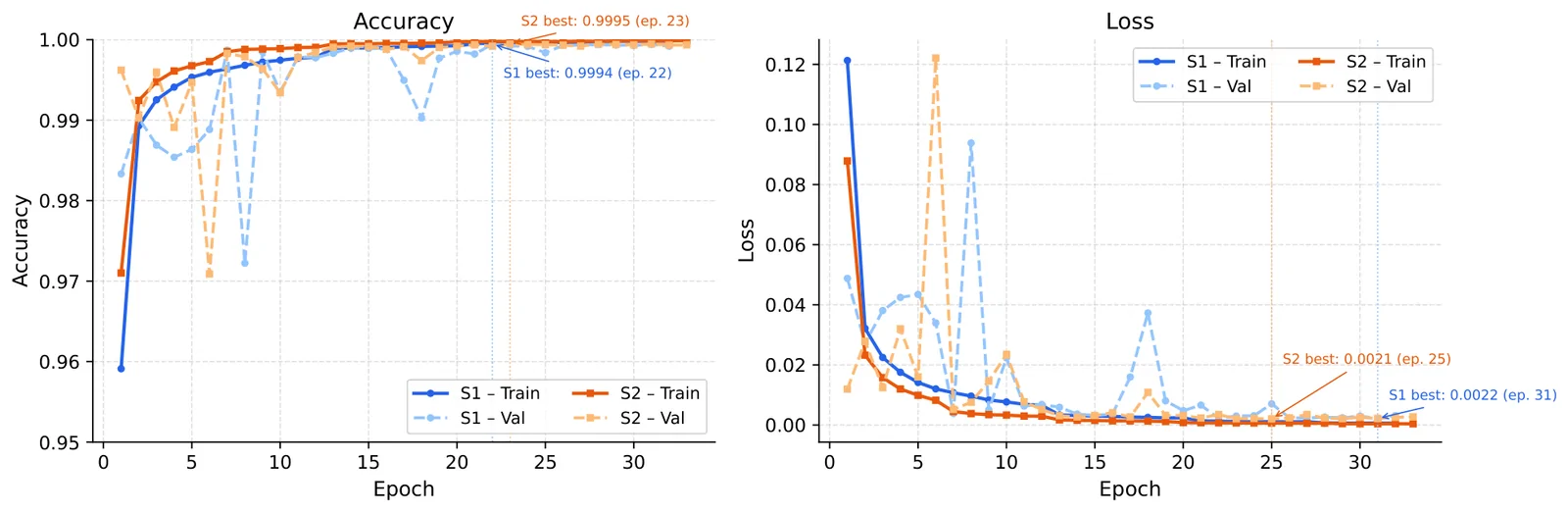
Combined training and validation accuracy (**left**) and loss (**right**) curves for CNN-1 (Sensor 1) and CNN-2 (Sensor 2).

The learning curves of both models are shown in Figure 3, which combines the accuracy and loss trajectories of CNN-1 (Sensor 1) and CNN-2 (Sensor 2) for direct comparison. Both branches indicate rapid and stable convergence, reaching near-perfect validation accuracy within the first 10 epochs. CNN-1 achieves a best validation accuracy of 99.94% (epoch 22), while CNN-2 attains 99.95% (epoch 23); the two branches follow, closely overlapping trajectories throughout training, confirming comparable convergence behavior despite being trained independently on signals from different sensor locations. The absence of overfitting, together with the low and stable validation loss (0.0022 and 0.0021 for CNN-1 and CNN-2, respectively), confirms that each branch independently learns a discriminative representation of fault signatures from its respective sensor position.

**Figure 4:**
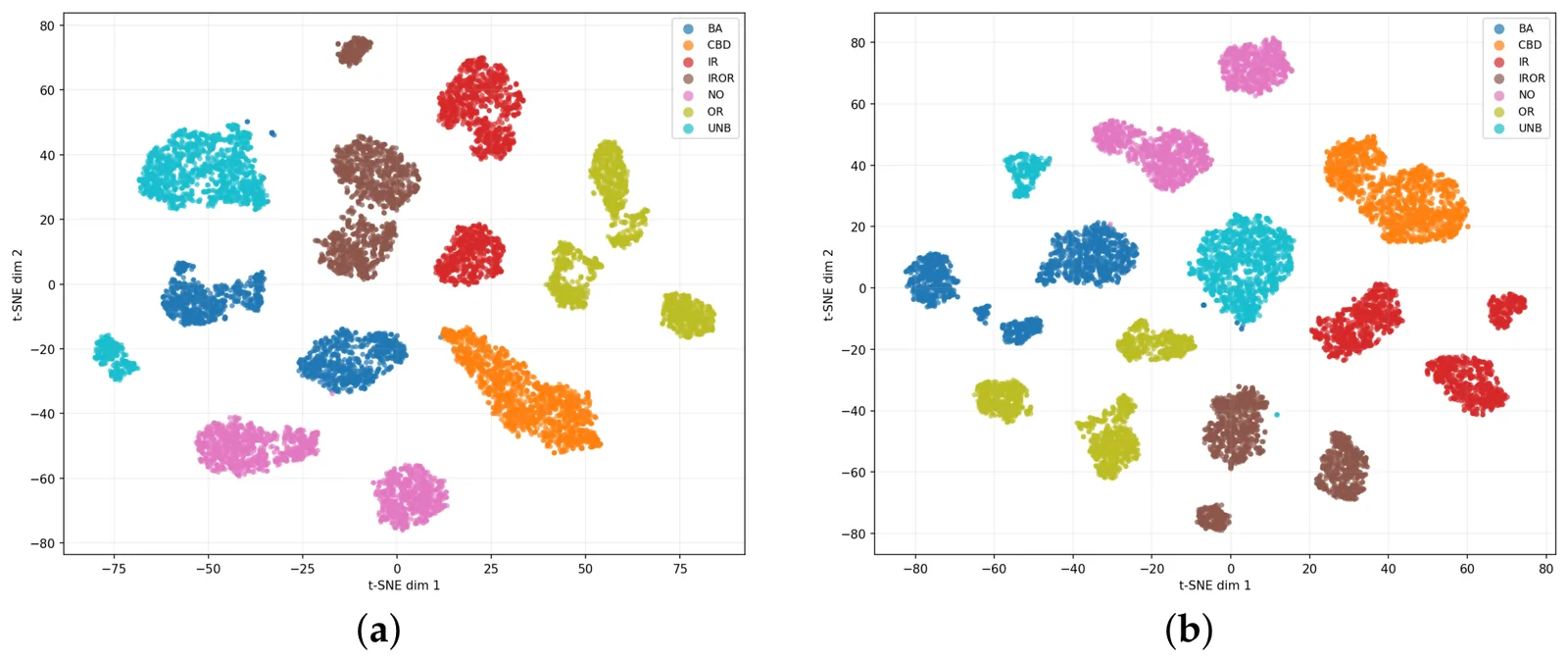
t-SNE visualization of feature embeddings extracted from the Dense(128) ReLU layer of (**a**) CNN-1 and (**b**) CNN-2. Classes: NO = healthy; BA = ball fault; OR = outer race fault; IR = inner race fault; CBD = combined defect; IROR = combined inner/outer race fault; UNB = shaft unbalance.

The t-distributed stochastic neighbor embedding (t-SNE) visualizations in Figure 4a,b, corresponding to CNN-1 and CNN-2, respectively, reveal well-separated and compact clusters across all seven fault classes (BA, CBD, IR, IROR, NO, OR, and UNB), confirming effective class-discriminative feature learning directly from raw vibration signals.

### 4.2. Benchmark of Fusion Strategies

#### 4.2.1. Performance Under Baseline Conditions

Under baseline conditions, the acquired signals are in their baseline laboratory noise conditions with no artificial added noise. Table 3 reports the classification performance of all six fusion techniques with the two individual CNN branches, which are averaged over five independent training rounds (mean ± std). All approaches achieve an average recognition accuracy above 99.7%, reflecting the high discriminative quality of both sensor channels; this is expected given that the synchronized, high-sensitivity accelerometers mounted directly on the bearing housings operate in a controlled, comparatively low ambient-noise laboratory environment, in which localized faults are clearly distinguishable in the raw waveform (Figure 2) and are readily separated by the deep CNN model. Consequently, all six fusion rules and both individual CNN branches fall within a narrow 99.78–99.82% band (Table 3), which is a spread smaller than the run-to-run standard deviation reported for most methods (0.04–0.14 percentage points). Although the differences are numerically small, a consistent direction is nonetheless observable: Max rule, Average rule, Dempster–Shafer, Majority Voting, and Weighted Sum match or exceed the better-performing individual branch (Sensor 2, 99.81%), while Stacking falls slightly below it. We interpret this as a small but repeatable fusion effect rather than as evidence that any one strategy is clearly superior under such low ambient-noise conditions; the differences reflect a ceiling effect in which both individual branches are already near-optimal, leaving negligible residual error for fusion to correct.

This ceiling effect indicates that the baseline scenario, while useful as a sanity check confirming that fusion does not degrade performance when the underlying classifiers are already highly reliable, is not sufficiently challenging to differentiate between fusion strategies. Real industrial environments expose sensors to electromagnetic interference, mechanical vibration from neighboring equipment, and variable operating conditions that produce ambient noise levels well beyond this controlled laboratory bench. The additive-noise experiment presented in the next subsection is designed to emulate this more realistic, diagnostically demanding regime, in which the individual branches and fusion strategies are shown to diverge substantially.

**Figure 5:**
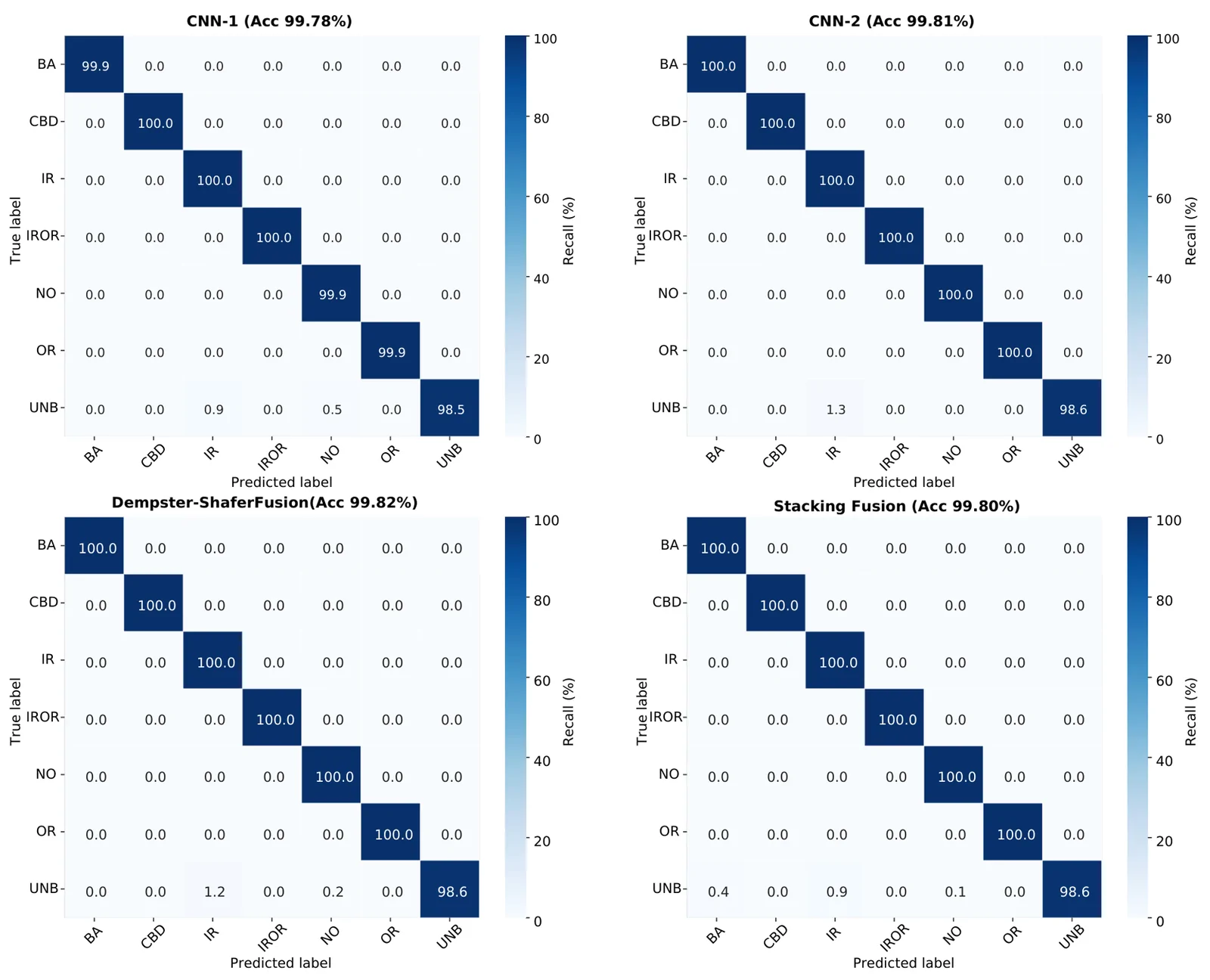
Confusion matrices for CNN-1, CNN-2, Dempster–Shafer fusion, and Stacking fusion under baseline conditions.

The confusion matrices in Figure 5 related to CNN-1, CNN-2, Dempster–Shafer, and Stacking techniques reveal only minor misclassifications, which are concentrated mainly between the UNB, BA, and IR classes. These are slightly reduced through decision fusion, particularly under the Dempster–Shafer combination.

#### 4.2.2. Statistical Significance Analysis

To assess whether the accuracy differences reported in Table 3 reflect statistically robust effects, we complement the five-round mean ± std comparison with a within-run pairwise statistical analysis on a representative test run. For each pair of methods, McNemar’s test compares their predictions on the same test samples and asks whether one method corrects the other’s mistakes more often than the reverse, which is a stronger test than comparing overall accuracy alone, since two methods can reach similar accuracy while disagreeing systematically on which samples were identified correctly. For a comparison “A vs. B,” $n_{10}$ and $n_{01}$ denote the number of samples on which only A, respectively only B, is correct; $n_{10}>n_{01}$ indicates that A outperforms B on the disputed samples. We use the exact binomial form of the test when the number of discordant pairs is small and the chi-squared (continuity-corrected) form otherwise, and we apply a Holm–Bonferroni correction across all pairwise comparisons among the eight evaluated methods to control the family-wise error rate.

As iilustrated in Table 4, both individual sensing branches differ significantly from every fusion strategy (Holm-corrected $p<4\times 10^{-7}$ throughout) even though the raw accuracy gaps are small (0.03–0.05 percentage points, cf. Table 3): several comparisons show an almost entirely one-sided error pattern (e.g., Sensor 1 vs. Max Rule, $n_{10}=0$, $n_{01}=55$), which is what drives the extreme significance despite the modest accuracy difference. Sensor 1 and Sensor 2 are not significantly different from each other (p=0.108), and no pairwise comparison among the fusion strategies reaches significance (p=1.000 throughout), indicating that once either branch is incorporated into a fusion rule, all fusion strategies perform statistically indistinguishably from one another at this near-ceiling accuracy level. Differentiating between fusion strategies therefore requires more challenging conditions, as explored in the additive-noise analysis of Section 4.2.3, or a more discriminative criterion than raw accuracy, motivating the conflict-coefficient-based analysis of Section 4.3.

#### 4.2.3. Robustness Under Additive Noise

To assess robustness under degraded signal conditions, white Gaussian noise was added to the test signals at SNR levels ranging from −2 dB to 40 dB with the signal-to-noise ratio defined as

(15) $${\mathrm{SNR}}_{\mathrm{dB}}=10\log_{10}\left(\frac{P_{\mathrm{signal}}}{P_{\mathrm{noise}}}\right).$$

**Figure 6:**
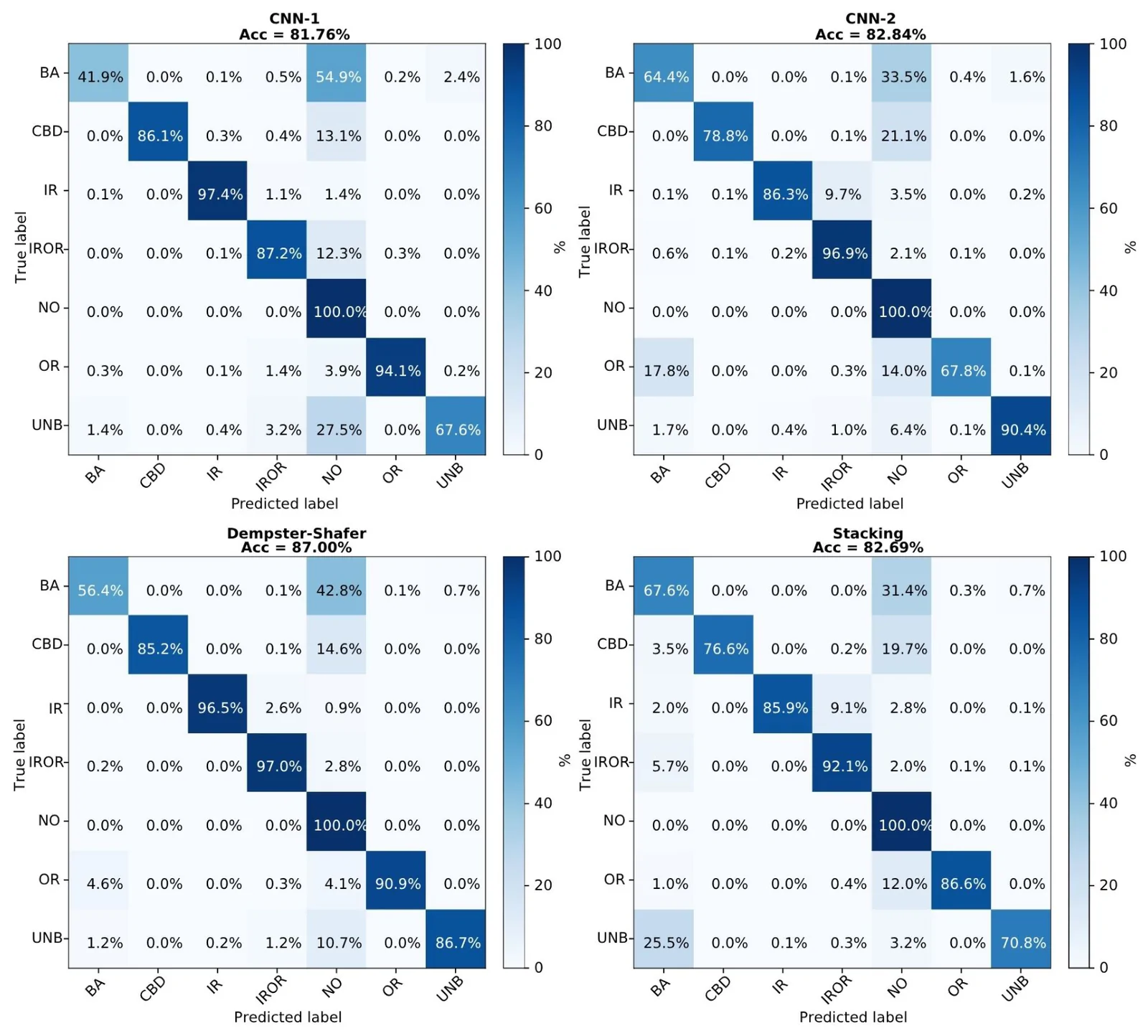
Confusion matrices at 10 dB SNR for CNN-1, CNN-2, Dempster–Shafer fusion, and Stacking.

**Figure 7:**
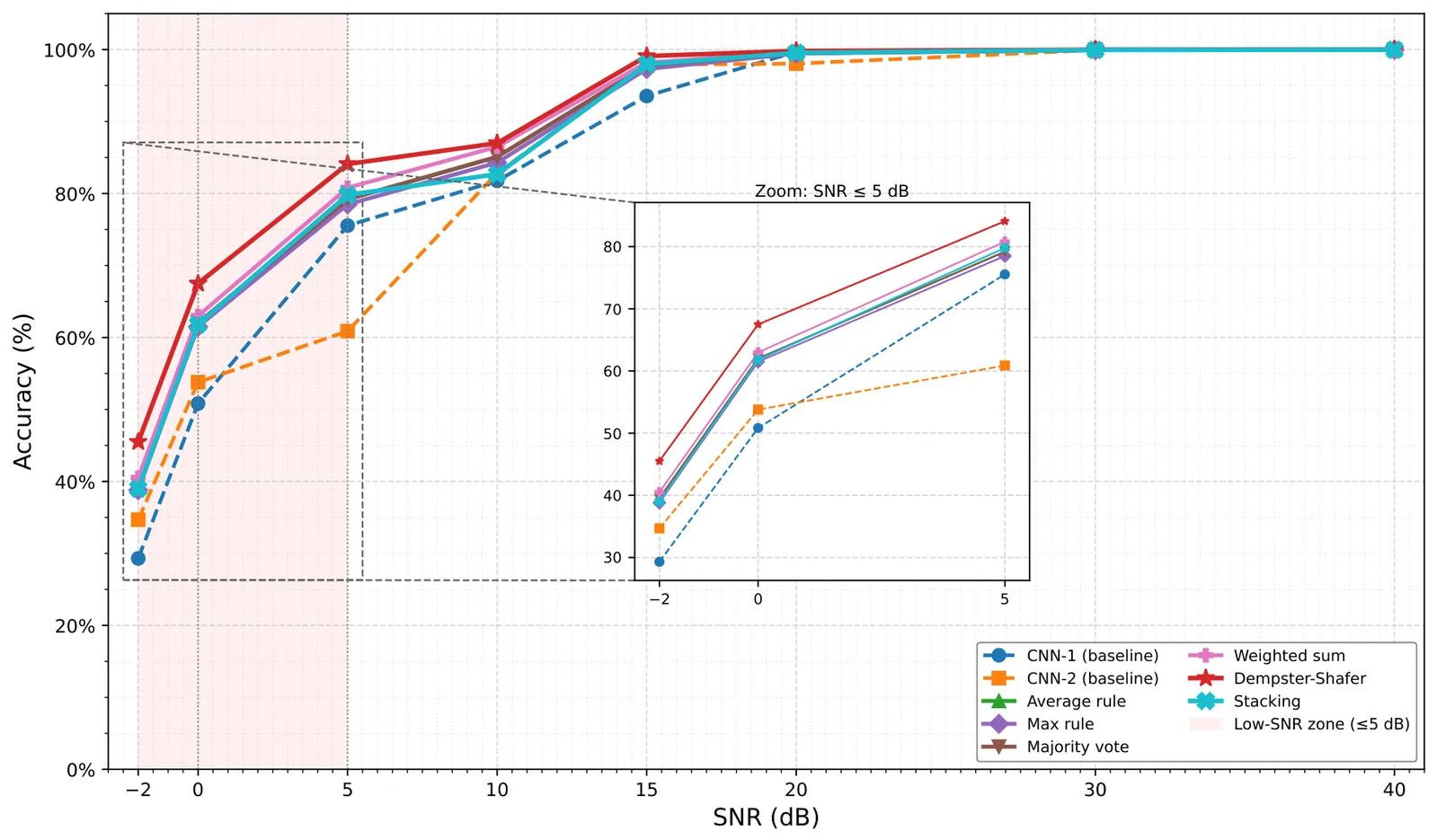
Accuracy of the fusion strategies and baseline CNNs as a function of the SNR noise level. The shaded region (≤5 dB) denotes the low-SNR zone, while the inset provides a magnified view of this region to facilitate a detailed comparison of the fusion strategies under severe noise conditions.

Figure 6 shows confusion matrices at 10 dB for both baseline CNNs and the two best-performing fusion strategies, and Figure 7 reports accuracy across the full SNR sweep.

In the low-SNR regime (≤5 dB), the individual branches degrade substantially, as highlighted by the zoomed inset in Figure 7. At −2 dB, CNN-1 and CNN-2 fall to approximately 29.3±1.8% and 34.7±1.6%, respectively. The fusion methods consistently improve upon the best individual CNN, although the magnitude of improvement depends on the fusion rule. At −2 dB, Average, Max, Majority Vote, Weighted Sum, and Stacking provide gains of 4.81, 4.11, 4.81, 5.81, and 4.31 percentage points, respectively, while Dempster–Shafer fusion achieves the highest accuracy of 45.5±1.7%, corresponding to a gain of 10.81 percentage points over the best baseline. At 0 dB, Dempster–Shafer further improves to 67.5%, exceeding the best baseline (53.79%) by 13.71 percentage points, while at 5 dB, it reaches 84.1% with an 8.55-percentage-point gain over CNN-1 (75.55%). These results demonstrate, particularly in the zoomed low-SNR region, that Dempster–Shafer fusion provides the most effective robustness against severe noise by exploiting complementary and uncertain evidence from the two CNN branches.

At 10 dB, both baselines exhibit their primary weakness on the Ball fault (BA) class, with a large share of samples misclassified as Normal (NO), suggesting that the subtle impulsive signature of ball defects is particularly sensitive to noise corruption; the CBD and UNB classes also show notable confusion. Dempster–Shafer fusion (87.00±1.1%) partially alleviates these confusions, the IR, IROR, NO, and OR classes all benefit from evidence combination, though BA remains challenging, with 42.8% of samples still misclassified as NO. Stacking (82.69±1.4%) reaches similar overall accuracy to the individual CNNs at this SNR (CNN-1: 81.76±1.5%; CNN-2: 82.84±1.4%) but distributes errors more evenly across classes, avoiding the strong class-specific failure seen in the baselines. This gap relative to Dempster–Shafer fusion stems from how each method generalizes to unseen noisy conditions. The SVM meta-learner is trained only on clean-signal validation probabilities and therefore learns a fixed decision boundary specific to that region of the probability space; under noise, the joint CNN-1/CNN-2 probability vectors shift into regions the meta-learner never observed during training, which is a distribution shift that a fixed kernel boundary handles poorly. Dempster–Shafer fusion, by contrast, is a parameter-free algebraic combination rule that requires no training and applies identically to any input probability distribution, which explains its more consistent advantage across the full SNR range.

Beyond 15 dB, both fusion strategies and individual CNNs converge toward near-perfect accuracy, confirming that the diagnostic task is well conditioned under low noisy conditions. Taken together, these results confirm that evidence-based fusion provides a meaningful robustness advantage over individual classifiers and simpler combination rules, particularly in the practically challenging low-SNR regime.

For the multi-location fault task, Table 5 reports recognition performance on the multi-location IROR condition, where each sensor captures a distinct, spatially localized fault and the branches are expected to be most complementary. Under baseline conditions, all methods reach 100% recall, leaving no method to discriminate. At 10 dB SNR, CNN-1 (Sensor 1) drops to 87.2% while CNN-2 (Sensor 2) reaches 96.9%; Dempster–Shafer fusion attains 97.0%, matching the stronger branch and improving nearly 10 points over the weaker one, while Stacking (92.1%) falls between the two. Fusion thus proves most valuable precisely when branches see different fault signatures without falling below the better-performing individual sensor.

The additive Gaussian noise model used here offers a controlled, single-parameter (SNR) benchmark that is standard in the fault-diagnosis literature, but it does not capture all disturbance types found in industrial settings, such as impulsive transients or gradual sensor drift. Impulsive noise, being non-stationary, could push branch confidences into the high-conflict regime identified in Section 4.3 more abruptly than stationary noise does, while sensor drift would degrade one branch asymmetrically rather than uniformly, which is a scenario that the fixed-weight rules evaluated here do not explicitly account for. Extending this benchmark to such non-Gaussian and field-recorded disturbances is an important direction for future work.

#### 4.2.4. Statistical Significance Analysis Under Noisy Conditions (5 dB SNR)

To evaluate whether the robustness advantage of Dempster–Shafer fusion observed under additive white Gaussian noise is statistically significant, we apply the same McNemar-based analysis (Section 4.2.2) to a representative degraded-SNR operating point (5 dB), where the two sensing branches exhibit strongly asymmetric reliability. Pairwise significance is assessed using McNemar’s test with Holm–Bonferroni correction across the 28 pairwise comparisons among the eight evaluated methods. As the statistical tests are conducted at the segment level, where overlapping windows from the same recording are not strictly independent, the reported *p*-values should be interpreted as strong directional evidence of relative performance rather than exact probabilities; the consistency of effect direction across all fusion-vs-baseline comparisons, together with the large accuracy margins reported in Figure 7, supports the robustness conclusion independent of the exact *p*-value magnitude.

At 5 dB SNR, nearly every pairwise comparison reaches statistical significance after Holm correction. Among the fusion-vs-fusion comparisons, 15 of 15 pairs are significant ($p\leq 1.8\times 10^{-3}$), which is a marked departure from the baseline (noise-free) condition, where no fusion-vs-fusion pair was significant. For a pairwise comparison labeled “A vs. B,” $n_{10}$ denotes the number of test samples on which method A is correct and method B is incorrect, while $n_{01}$ denotes the number of samples on which A is incorrect and B is correct. Consequently, $n_{10}>n_{01}$ indicates that A outperforms B on the discordant samples and vice versa. For example, for the comparison “Max rule vs. Dempster–Shafer” ($n_{10}=157$, $n_{01}=746$), the Max rule is correct while Dempster–Shafer is wrong on only 157 samples, whereas Dempster–Shafer is correct while the Max rule is wrong on 746 samples.

Dempster–Shafer fusion also wins the majority of discordant samples against every other fusion rule (all comparisons Holm-corrected, $p\leq 1.8\times 10^{-3}$, Table 6). Dempster–Shafer fusion’s discordant-pair pattern is consistently favorable against the simpler combination rules with all comparisons expressed as (DS-correct, X-wrong) vs. (DS-wrong, X-correct): vs. Max Rule (746 vs. 157), vs. Majority Voting (367 vs. 173), vs. Weighted Sum (369 vs. 163), and vs. Stacking (4104 vs. 1358). In every case, DS wins the majority of discordant samples, confirming its statistically robust advantage over both individual branches and the competing fusion rules.

### 4.3. Confidence Complementarity and Fusion Challenges

**Figure 8:**
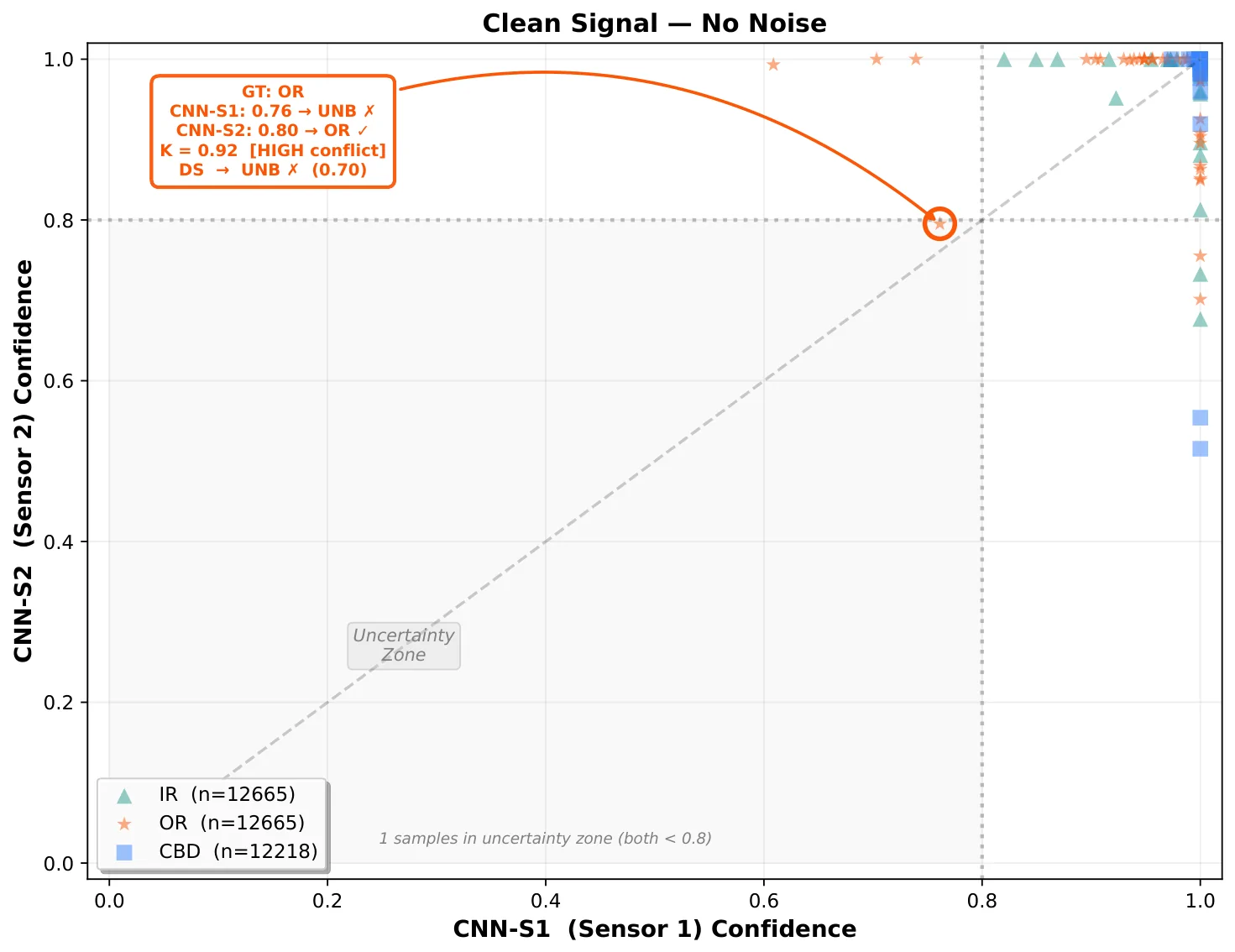
Per-sample confidence scatter of CNN-1. ✓/× denote correct/incorrect predictions in the annotated examples.

**Figure 9:**
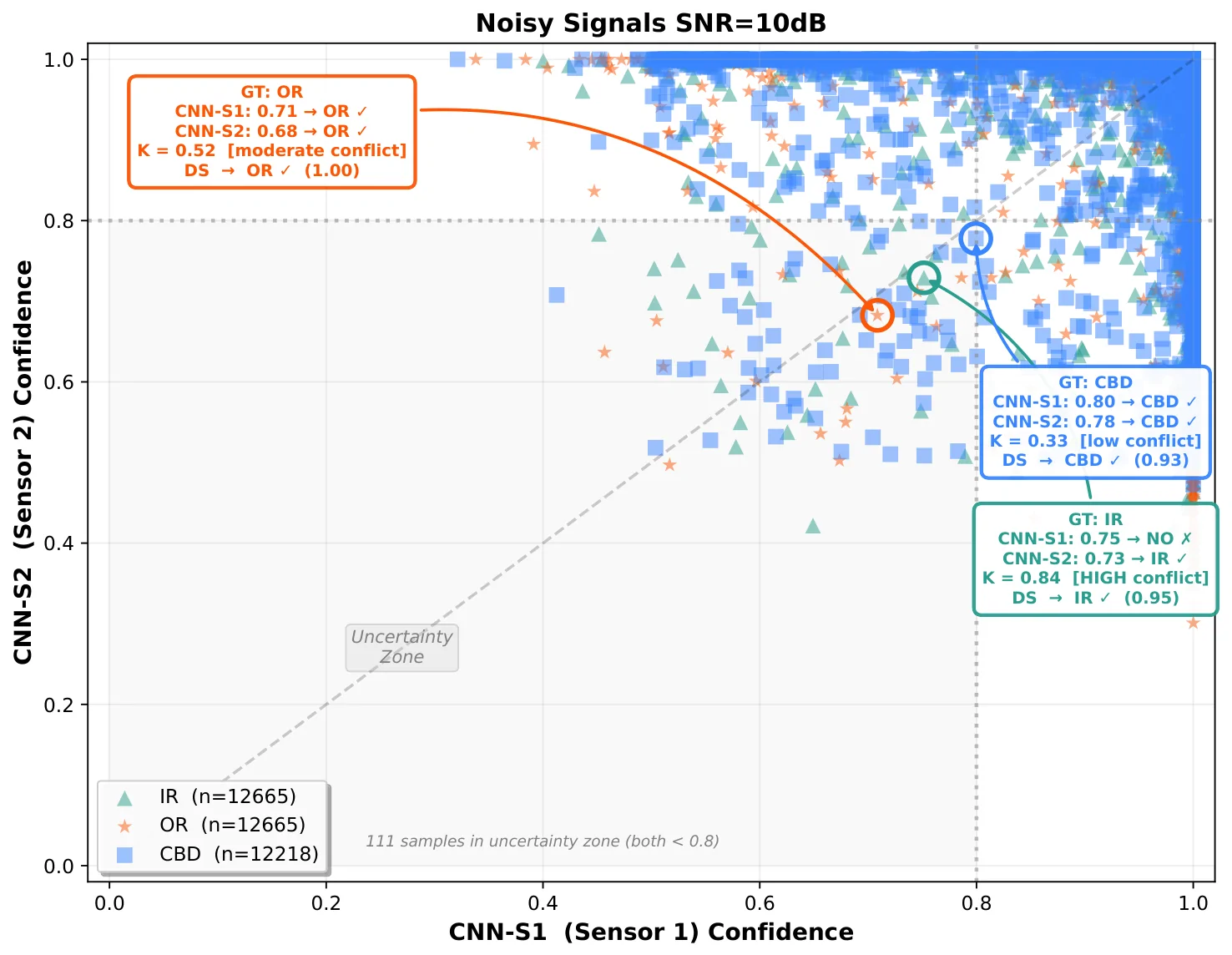
Per-sample confidence scatter of CNN-2. ✓/× denote correct/incorrect predictions in the annotated examples.

To complement the accuracy analysis, an examination of the per-branch softmax confidence scores is conducted to provide an interpretable framework of how the DST fusion behaves at the individual prediction level. Representative examples are provided for the IR, OR, and CBD fault classes. For each test sample, the confidence scores of CNN-1 and CNN-2 are jointly visualized in a two-dimensional confidence space, where the dashed lines at 0.8 delimit the *uncertainty zone*—the region in which neither branch produces a sufficiently confident prediction. The analysis is performed under two conditions: baseline condition signals and noisy signals at moderate noise level SNR=10 dB, as shown in Figure 8 and Figure 9.

Under baseline clean conditions (Figure 8), samples from all three classes (IR, OR, CBD) are tightly concentrated in the high-confidence region with only a single sample falling in the uncertainty zone. The annotated case reveals a failure mode of DS under high inter-sensor conflict (K=0.92): although CNN-2 correctly identifies the OR fault, the strong disagreement with CNN-1 causes DS to produce an incorrect prediction, illustrating the well-known sensitivity of Dempster’s rule to highly contradictory evidence sources. At SNR=10 dB (Figure 9), the confidence landscape degrades noticeably: the uncertainty zone population rises from 1 to 111 samples, and the point cloud spreads across the mid-confidence region. The three annotated cases demonstrate the contrasting behaviors of DS fusion under varying conflict levels. When both branches agree, even at moderate confidence, DS consolidates their evidence and produces a correct, high-belief prediction (OR case, K=0.52, belief =1.00; CBD case, K=0.33, belief =0.93). More importantly, when one branch is misclassifying while the other retains a correct but uncertain prediction (IR case, K=0.84), DS still recovers the correct diagnosis with a belief of 0.95, highlighting its capacity to arbitrate between conflicting sources rather than defaulting to the more confident but incorrect branch.

These observations highlight two core challenges of decision-level fusion: (i) identical coordinates can conceal opposite CNN predictions, making the conflict coefficient *K* an essential diagnostic indicator that goes beyond the raw confidence margin; and (ii) Dempster–Shafer fusion under high conflict (K≥0.70) can produce overconfident incorrect decisions. This stems directly from setting $m_{i}\left(\Theta \right)=0$ when building the BBA from softmax outputs with no mass reserved for residual ignorance; here, *K* is driven entirely by the branches’ full probability distributions, and dividing by 1−K sharply inflates the remaining mass regardless of its reliability, as in the K=0.92 case above. A confidence-derived $m_{i}\left(\Theta \right)$ (e.g., proportional to $1-\max_{k}p_{i,k}$) could let overconfident-but-wrong branches yield ground preemptively, reducing *K* at the cost of lower belief in genuine high-confidence agreements. This trade-off motivates the need for a conflict-aware rejection mechanism that abstains or defers to the more reliable branch when *K* exceeds a learned threshold.

## 5. Conclusions

This paper presented a systematic comparative benchmark of six decision-level fusion strategies, i.e, Max, Average, Majority Voting, Weighted Sum, Dempster–Shafer, and Stacking, for combining two independently trained CNN branches in multi-sensor bearing fault diagnosis. While all strategies performed comparably under baseline conditions, their behavior diverged sharply under noisy conditions where the Dempster–Shafer fusion consistently outperformed the individual CNN branches and the simpler fusion rules in the low-SNR regime, confirming that evidence-based combination offers a robustness advantage precisely where single-sensor diagnosis and simple fusion approaches fall short.

This advantage was explained by the confidence and conflict-coefficient analysis, which showed that fusion succeeds when branches provide complementary evidence but can produce overconfident errors when inter-branch conflict is high (K ≥ 0.70), which make the conflict coefficient a useful diagnostic and interpretable component beyond raw classification confidence. The benefit was also location-dependent: on the multi-location fault (IROR), fusion tracked the stronger branch under noise rather than exceeding it, protecting against the weaker branch’s degradation. Together, these results indicate that fusion helps most when the sensors truly complement each other.

These findings motivate conflict-aware rejection or the conflict threshold adaptive mechanism that abstains from fusion, or defers to the more reliable branch, when conflict exceeds a threshold. Rather than a fixed value, this threshold could be learned on validation data or replaced by a continuous weighting of the fused decision as a function of *K*, avoiding the abruptness of a binary accept/reject rule.

The applicability of this benchmark is also bounded by its scope. All results are obtained with M=2 branches; extending decision-level fusion to more sensors is expected to raise the computational cost of Dempster’s rule and to complicate the interpretation of pairwise conflict, since *K* would need to be generalized beyond a single scalar per fusion. Likewise, the multi-location case considered here (IROR) involves exactly two co-occurring, spatially separated faults; systems exhibiting three or more simultaneous defects, or defects overlapping on a single sensor, remain untested and may challenge the branch-independence assumption underlying decision-level fusion.

A further limitation concerns the experimental setting itself: all results were obtained on a laboratory test bench which, despite spanning three rotational speeds (10, 15, 20 Hz) and two load conditions (no load, 5 kg), lacks the structural resonances and non-stationary disturbances of real industrial operation, so the reported results may not transfer directly to field conditions. Embedding the framework within a transfer learning setting is a promising way to close this gap: since each CNN branch is trained independently and fusion is applied only after each branch produces its output, a branch could be domain-adapted to real industrial data without retraining the other branch or the fusion rule, preserving the framework’s modularity and low deployment overhead. The conflict coefficient K could further serve as an indicator of domain shift during this adaptation. Extending the framework beyond two sensor sources and validating it, potentially through this transfer learning route, on real multi-location industrial systems, such as axle-bogie train bearings, under varied operational and multi-fault conditions, remain important directions for future work.

## Figures and Tables

**Table 1:** Dataset composition and bearing fault locations (60:20:20 train/val/test split).

Condition	Label	Bearing 1	Bearing 2	Direct-Fault	Total
		State	State	Sensor(s)	Signals
Healthy	NO	Healthy	Healthy	–	540
Ball fault	BA	Ball fault	Healthy	Sensor 1	540
Outer race fault	OR	Outer race	Healthy	Sensor 1	540
Inner race fault	IR	Inner race	Healthy	Sensor 1	540
Combined defect	CBD	BA+OR+IR	Healthy	Sensor 1	540
Inner+outer race	IROR	Outer race	Inner race	Sensors 1 & 2	540
Unbalance	UNB	–	–	Sensors 1 & 2	540
Total					3780
Train (60%)					2268
Val/Test (20%/20%)					756/756

**Table 2:** The 1D-CNN architecture.

Block	Operations	Output
1	Conv1D(32) → BN → ReLU → MaxPool	(256, 32)
2	Conv1D(64) → BN → ReLU → MaxPool	(64, 64)
3	Conv1D(128) → BN → ReLU → MaxPool	(16, 128)
4	Conv1D(256) → BN → ReLU → MaxPool	(4, 256)
–	GlobalAvgPool → Dense(128) → Dropout → Softmax(7)	(7)
Total params: 169,063		

**Table 3:** Comparison of 5 rounds of recognition results under baseline conditions for different fusion techniques (mean ± std).

Technique	Accuracy (%)	F1-Score (%)	Precision (%)
Max Rule	99.82±0.1	99.81±0.09	99.83±0.1
Dempster–Shafer	99.82±0.08	99.81±0.07	99.83±0.07
Majority Voting	99.82±0.06	99.81±0.06	99.83±0.07
Weighted Sum	99.82±0.12	99.81±0.1	99.83±0.1
Average Fusion	99.82±0.08	99.81±0.08	99.83±0.09
Sensor 2	99.81±0.04	99.80±0.05	99.82±0.07
Stacking	99.80±0.13	99.80±0.13	99.81±0.14
Sensor 1	99.78±0.11	99.77±0.12	99.78±0.14

**Table 4:** McNemar pairwise significance testing under baseline (noise-free) conditions (Holm-corrected, α=0.05) grouped by comparison type.

Comparison Type	# Pairs	*p*-Range (Holm-Corrected)	Significant?
Sensor 1 vs. Sensor 2	1	p=0.108	No
Sensor 1 vs. fusion method	6	$\left[6.9\times 10^{-13},\;9.9\times 10^{-13}\right]$	Yes (all 6)
Sensor 2 vs. fusion method	6	$\left[4.9\times 10^{-8},\;3.9\times 10^{-7}\right]$	Yes (all 6)
Fusion method vs. fusion method	15	p=1.000	No (all 15)

**Table 5:** Per-branch and fused recognition performance on the multi-location IROR condition under baseline and 10 dB SNR conditions.

Condition	CNN-1	CNN-2	Dempster–Shafer	Stacking
Baseline	100.0%	100.0%	100.0%	100.0%
10 dB SNR	87.2%	96.9%	97.0%	92.1%

**Table 6:** McNemar pairwise significance testing at 5 dB SNR (Holm-corrected, α=0.05), grouped by comparison type.

Comparison type	# Pairs	*p*-Range (Holm-Corrected)	Significant?
Sensor 1 vs. Sensor 2	1	$p=8.3\times 10^{-272}$	Yes
Sensor 1 vs. fusion method	6	$p<10^{-300}$	Yes (all 6)
Sensor 2 vs. fusion method	6	$p\in [9.7\times 10^{-276},\;1.1\times 10^{-69}]$	Yes (all 6)
Fusion method vs. fusion method	15	$p\in [10^{-301},\;1.8\times 10^{-3}]$	Yes (15/15)

*Note:* Corresponding accuracy values for all methods at this SNR level are reported in Figure 7.

## Data Availability

Data will be made available upon request.

## Abbreviations

The following abbreviations are used in this manuscript:

CNN	Convolutional Neural Network
1D-CNN	One-Dimensional Convolutional Neural Network
DLF	Decision-Level Fusion
DST	Dempster–Shafer Theory
BBA	Basic Belief Assignment
SNR	Signal-to-Noise Ratio
BN	Batch Normalization
ReLU	Rectified Linear Unit
SVM	Support Vector Machine
t-SNE	t-distributed Stochastic Neighbor Embedding
GPU	Graphics Processing Unit
BA	Ball Fault
OR	Outer Race Fault
IR	Inner Race Fault
CBD	Combined Defect
IROR	Inner and Outer Race Fault (multi-location)
UNB	Unbalance
NO	Normal (Healthy) Condition
MFS	Machinery Fault Simulator
CSI	Complex Systems and Interactions (Laboratory)
